# The World-Wide Adaptations of Diabetic Management in the Face of COVID-19 and Socioeconomic Disparities: A Scoping Review

**DOI:** 10.7759/cureus.31911

**Published:** 2022-11-26

**Authors:** Jaafar Abou-Ghaida, Annalia Foster, Sarah Klein, Massah Bassie, Khloe Gu, Chloe Hille, Cody Brown, Michael Daniel, Caitlin Drakeley, Alek Jahnke, Abrar Karim, Omar Altabbakh, Luzan Phillpotts

**Affiliations:** 1 College of Medicine, Nova Southeastern University Dr. Kiran C. Patel College of Osteopathic Medicine, Clearwater, USA; 2 Family Medicine, Nova Southeastern University Dr. Kiran C. Patel College of Osteopathic Medicine, Davie, USA

**Keywords:** socioeconomic factors, management, treatment, disparities, diabetes, covid-19

## Abstract

Diabetes is an increasingly prevalent chronic disease throughout the world. It is imperative for patients to have access to reliable treatment and resources in order to avoid long-term complications. Economic and social factors contribute to the accessibility of these resources and have a direct impact on diabetes management. Socioeconomic status (SES) presents challenges to diabetic management due to financial and geographical access to care, medications, educational resources, healthy food options, and physical activity. The coronavirus (COVID-19) pandemic exacerbated these challenges, especially during the height of lockdowns. Therefore, it is important to gain insight into how the pandemic challenged diabetes management, taking into consideration socioeconomic disparities. The objective is to assess how the COVID-19 pandemic has impacted the care of chronic diabetic patients internationally and determine how these outcomes vary between patients of different socioeconomic classes. The following study was designed as a scoping review and utilized PubMed, EMBASE, CINAHL, and Web of Science. A Boolean search strategy combined search terms as follows: (((COVID-19) AND (diabetes)) AND ((socioeconomic factors) OR (social inequality OR standard of living))) AND (treatment OR management). Inclusion criteria included studies addressing diabetic patients, socioeconomic variables (income, occupation, level of education, and ethnicity), glycemic control, and degree of access to quality healthcare. Studies exploring the pathophysiology of COVID-19 or diabetes mellitus were excluded. In addition, studies were chosen between the years 2020 and 2022. The search resulted in 214 articles. The full-text assessment was then conducted on the remaining 67 articles. After screening for eligibility and relevance, 19 articles were retained for this review. The results of this study indicate that 8 out of the 18 studies revealed worse outcomes for those with diabetes mellitus and concomitant COVID-19 infection. Patients with diabetes were more likely to be hospitalized and represent a larger percentage of COVID-19 fatalities. In addition, patients with diabetes and co-morbid COVID-19 infection were more likely to have a higher hemoglobin A1c (HbA1c), belong to a lower SES, and have worse glycemic control due to pandemic-associated lockdown. In order to combat the effects of the pandemic, many countries created novel and innovative management strategies. Overall, there are positive and negative effects from the pandemic on diabetic management strategies. This scoping review identified successes in diabetic treatment under pandemic conditions and areas that need optimization. The successful adaptations of many nations convey the capacity for new policy implementation to care for diabetic patients regardless of SES.

## Introduction and background

Diabetes mellitus, a chronic condition that impacts glucose metabolism, is an increasingly prevalent condition globally, which if not properly managed can result in serious complications. Management requires that patients develop a reliable treatment plan with their practicing clinicians and have access to resources to allow for proper adherence on a daily basis in an effort to maintain proper glycemic control. Meanwhile, the ongoing coronavirus disease (COVID-19) pandemic has impacted how patients with diabetes manage their condition. Financial constraints, food access, and medical supply shortages are just a few of the issues that have negatively impacted patients in the management of their diabetes [1]. This is problematic, as patients with diabetes are at a higher risk of poor health outcomes tied to COVID-19 infection [2].

Diabetes management during the COVID-19 pandemic changed as a result of lockdowns, the high risk accompanying co-morbid complications such as acute infections, incorporation of telemedicine, and socioeconomic status (SES) factors. One study noted that education and occupation were two of the more significant associations with medicine accessibility during the COVID-19 pandemic [3]. SES has a profound impact on diabetic management due to varying degrees of financial and geographical access to care, medications, educational resources, healthy food options, and physical activity. As such, this scoping review seeks to assess how the management of diabetes has changed during the COVID-19 pandemic among patients of varying SES.

## Review

Methods

**Figure 1:**
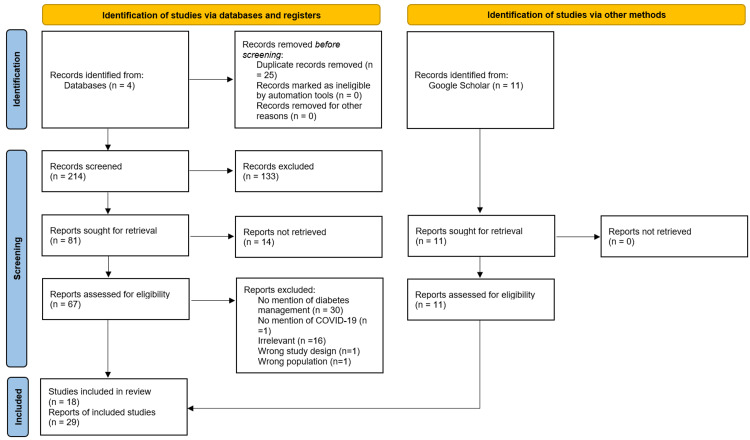
PRISMA Chart Detailing Abstract and Full Article Review Process

The following scoping review utilized PubMed, CINHAL, EMBASE, and Web of Science to find relevant articles that fit the protocol’s inclusion criteria. In addition, google scholar was used to find additional articles. Studies assessing patients with diabetes, socioeconomic variables (income, occupation, level of education, and ethnicity), glycemic control, and degree of access to quality healthcare were included. Any study failing to mention the impact of COVID-19 on diabetes management or prioritized predictors of COVID-19 mortality instead were not included, except for background information purposes only. In addition, studies exploring the pathophysiological course of COVID-19 or diabetes mellitus were excluded. We used a Boolean search strategy that combined search terms as follows: (((COVID-19) AND (diabetes)) AND ((socioeconomic factors) OR (social inequality OR standard of living))) AND (treatment OR management). In addition, the time frame filter was placed between the years 2020 and 2022. This search resulted in 214 articles (Figure 1). The first round of screening involved abstract screening and removed 133 articles by virtue of being irrelevant to the research topic. Sixty-seven articles were found using EndNote's find full-text feature of the 81 remaining articles. The second round of screenings filtered through these 67 articles and involved full-text assessment of the articles’ content. Thirty additional articles were excluded for failure to address diabetes management in the context of the COVID-19 pandemic, and 16 articles were excluded for being irrelevant to the topic at hand. The final database abstract and full article screenings yielded 18 articles. The google scholar screening, on the other hand, yielded 11 select articles. The total number of articles included in this scoping review was 29 (Figure 1).

Results

Diabetes, COVID-19, and SES

The ongoing COVID-19 pandemic has impacted patients with diabetes in many ways, especially as this population of patients is more susceptible to hospitalization and adverse outcomes associated with contracting the virus [4]. Diabetes is associated with poorer health outcomes among COVID-19 patients, with hyperglycemia during hospital stay being a major risk factor for death [5]. One study conducted in the United Kingdom found higher averages of hemoglobin A1c (HbA1c), body mass index (BMI), and C-reactive protein in hospitalized patients compared to non-hospitalized patients [6]. Another study found that an HbA1c level > 9 percent was identified as a strong predictor of severe COVID-19 infection and subsequent hospitalization [7]. Studies also revealed patients with poor blood sugar control (high HbA1c) and hyperglycemia on admission have a higher risk of adverse outcomes such as ICU admission, mechanical ventilation, and increased mortality, especially in cases of severe COVID-19 [8-11]. Therefore, practicing clinicians should assess treatment management and consider adjusting medications appropriately, and encourage lifestyle modification.

Even though the pathological mechanism of hyperglycemia and COVID-19 contributed to increased mortalities, numerous healthcare barriers for patients with diabetes were also seen to cause more severe COVID-19 disease. Many countries participated in lockdowns, which caused a decrease in access to healthcare providers and medical supplies. This led to the deterioration of glycemic control and increased rates of observed ketoacidosis due to poor diabetes management. Fear of infection also caused people to avoid healthcare facilities that were open. One study based in India utilized predictive models that revealed the duration of lockdown to be directly proportional to the worsening of glycemic control in patients with diabetes and overall increased diabetes-related complications [12]. An increased load of diabetes-related complications has put an additional load on an overburdened public healthcare system. In addition, uncontrolled hyperglycemia and increased cardiovascular complications have the potential to increase the severity of COVID-19 infections in patients [13].

The pandemic has widened previously existing economic and health disparities, causing proper healthcare and diabetes management practices to become less attainable by patients of lower SES [14]. One study found that poverty level was associated with higher rates of hospitalization and death [15]. One of the most prominent mechanisms for this relationship is related to residents living in high-population density areas [16]. Living in such areas is associated with difficult social distancing and co-morbidities including obesity, which can lead to overall higher susceptibility to infection and mortality [16]. Studies based in the United States and Italy reinforced that notion by stating that living in poverty, severe housing problems, lack of English proficiency in either country, high population density, and diabetes prevalence are predictors of COVID-19 mortality [17,18]. Minority ethnic populations including African Americans are disproportionately affected by the inflammatory disease, which can cause a hyperactive immune response to infections such as COVID-19 [19]. In addition, African Americans and Hispanics were more likely to live in households containing health sector workers and workers unable to work remotely from home, respectively [20]. Overall, both diabetes and SES status are directly related to risks associated with COVID-19 infection and subsequent complications.

Lockdowns increased snacking habits and decreased physical activity, which resulted in weight gain, loss of glycemic control, and increased diabetic complications such as retinopathy, microalbuminuria, and proteinuria [21]. It was concluded that those who die from COVID-19 infection with co-morbid diabetes are usually those whose diabetes is not well controlled. As such, pandemic-inflicted restrictions and loss of healthcare access put diabetic patients at risk for severe COVID-19 infection. These barriers to care were exacerbated for those with lower SES. Access to medication and self-monitoring elements were restricted by lockdowns, but were also no longer affordable for many due to loss of employment. Even countries with universal healthcare required in-person prescription renewals every month, causing additional risk for exposure to COVID-19 infection and yet another barrier to access [21]. Persons of lower SES were often subjected to higher infection rates due to informal working conditions by finding jobs on the street, crowded living situations, and necessary daily market runs.

Diabetic Management

Measures were taken by several countries to combat the pandemic-induced decline in diabetic care management. Government agencies put in place longer prescriptions active for three months instead of one, free consultations with endocrinologists, guidelines on how to access certified internet-based medical services and medical supplies, and pharmacy maps with access to insulin, so hospital trips were minimized [21]. Telemedicine and tele-prescription tools were also highly utilized, but these tools also had their fair share of barriers. Telemedicine required internet access and technological equipment that was lacking in the residence of lower SES patients [21]. This suggested that individuals with diabetes and lower SES were more significantly impacted by the COVID-19 pandemic.

Increased attention on health among patients with diabetes could improve adherence to their care plan during the pandemic, especially as they are at a higher risk of adverse effects associated with COVID-19 [22]. Non-pharmacological recommendations for patients with diabetes changed during the pandemic to emphasize at-home exercise regimens, strict adherence to social distancing protocols due to increased COVID-19 risk, and stock-piling or online purchasing of glycemic testing supplies [23]. Medications that were trialed for COVID-19 treatment such as high-dose glucocorticoids and hydroxychloroquine therapy have glycemic-altering effects, and thus patients involved in these trials needed closer diabetic management [23].

Despite the evidence that diabetes and lower SES are linked to worse outcomes for COVID-19-infected patients, there is also some evidence that suggests that diabetes management has not changed in select parts of the world. Among type 1 diabetes mellitus patients residing in the United Kingdom, strict lockdowns during the early days of the pandemic were not associated with any change in glycemic control when flash glucose monitoring was utilized [24]. Another study showed that many patients with diabetes, including patients in wealthier zip codes, struggled to keep their glycemic ranges within normal limits for longer periods of time while stay-at-home orders were in place, putting them at greater risk for adverse outcomes associated with COVID-19 [25].

Discussion

Diabetes has worsened the clinical progression of COVID-19 in patients with comorbid conditions such as diabetes. A modelling study by Clark et al. mentions that an estimated 22% of the population has at least one underlying condition that could increase the risk of severe COVID-19 [26]. A study by de Souza et al. mentions that higher hospitalization was observed in the older populations owing to their multiple co-morbid statuses [27]. A study by Khunti et al. has tried to make the association that progression to severe COVID-19 may be attributed to being on diabetic medicine regimens such as alpha-glucosidase inhibitors and DPP inhibitors [28]. However, this study remained inconclusive (Table 1).

The COVID-19 pandemic undeniably brought an exhaustive number of changes to people's daily routines, major healthcare policies, and healthcare delivery. Patients with diabetes faced numerous barriers to their healthcare. However, the COVID-19 pandemic also changed the way healthcare was delivered, causing a shift to virtual medicine in both types of patients, with and without diabetes mellitus [29]. Virtual medicine increased in prevalence during the pandemic, and eliminated, or at least minimized, barriers to healthcare accessibility. If a patient could not afford gas or lives in a major healthcare desert, virtual medicine helped to overcome these barriers and provide patients with healthcare. For patients with diabetes, virtual changes during the pandemic prompted the increased utilization of advanced glucose monitoring systems (continuous glucose monitor (CGM)), which helped physicians more closely monitor patients using cloud-based technology. This enabled patients to make more informed decisions about their diabetes treatment regimen and increasing their awareness of how certain factors directly affect their glucose levels [29]. However, individuals who may not have access to technology, limited technology skills, or poor internet connection will experience minimal benefit from virtual medicine. This highlights the importance of improving access to care.

The evidence from these studies clearly suggests that patients with diabetes are more at risk for adverse outcomes, including death, secondary to COVID-19 infection (Table 1). The pandemic presented a challenge by compounding these risks, which potentially made diabetic management more difficult. Meanwhile, some evidence also indicates that the COVID-19 pandemic has not had a significant impact on how patients with diabetes manage their condition. As such, effective preventive medicine should not be complacent. Preventive medicine is a key component of a healthcare plan, and it should focus on exhausting available resources to make quality healthcare accessible, affordable, and beneficial to patients, despite their medical comorbidities or SES.

**Table 1:** Impact of COVID-19 and Socioeconomic Disparities on Diabetes Management

Table 1 Impact of COVID-19 and Socioeconomic Disparities on Diabetes Management					
Reference	Sample	Purpose	Methods (Design)	Results	Limitations
Khunti et al. [28]	Patients with type 2 diabetes registered in England between 1/1/2018 to 03/31/2019 and were alive on 02/16/2020. Sample size was 2,851,465.	Assess the relationship between glucose-lowering medications and COVID-19 mortality.	Observational cohort study	Minimal increase in COVID-19 mortality is seen in patients taking insulin, alpha-glucosidase inhibitors, and DPP inhibitors compared to other glucose-lowering medications. Difference is thought to be due to increased severity of disease in patients associated with increased risk of mortality.	Study did not account for frailty of the patient as this factor was not provided in the database used for the study. Data is likely confounded by indication for the medications used.
Nguyen et al. [17]	The study examined 159 counties within Georgia. Sample size was 17,286.	Determine the community variables associated with county-level COVID-19 cases, hospitalizations, and mortality.	Secondary data analysis data acquired from the 2020 County Health Rankings, the 2010 US Census, and the Georgia Department of Public Health COVID-19 Data was evaluated via multivariable linear regression models.	Four findings: The percentage of children living in poverty was found as the most significant predictor of COVID-19 rates. The percentage of severe housing problems appeared as another critical predictor associated with a higher risk of COVID-19 cases, hospitalization, and death rates. Regression analysis showed that the percentage of people not proficient in English was a strong positive predictor as well. Georgian counties diabetic prevalence varied from 5.6% to 32.7%, with the majority, 117 out of 159, above 11.4%, putting Georgia at a potentially higher risk for increased COVID-19 hospitalization and death rates.	Four limitations: Some results remained unexplained due to the involvement of multiple sites in collecting and interpreting the data. More work was needed on explaining racial and ethnic variables and how they contributed to COVID-19 cases and mortality. Testing numbers were not reported which limited the study's scope. Finally, data dates back to September 30, 2020 (more data has emerged since).
Campbell-Scherer et al. [14]	Families who worked with cultural brokers during the pandemic from September 21, 2020, through December 31, 2020.	To identify barriers to COVID-19 prevention and treatment among culturally diverse populations in Canada.	Cultural brokering is defined as linking people of differing cultural backgrounds for the purpose of producing positive change and minimizing conflict [14]. The researchers recorded narratives from families who worked with cultural brokers during the pandemic from September 21, 2020, through December 31, 2020. The sample size was small (16 women and 5 men), but they evaluated the narratives and categorized them into several domains.	They found that (1) financial insecurity, job loss, and inconsistent employment were all hampering COVID prevention and management, (2) more cultural brokers are needed in communities to effectively combat COVID, (3) prevention and management were being compromised by misinformation because of language barriers and cultural distance in the way community members receive and understand information, and (4) strong communication networks and solidarity are a strength that could aid in combatting the pandemic.	Small sample size (16 women and 5 men)
Oliva et al. [18]	Twenty Italian regions were studied.	Identify the determinants of population health involved in the cross-regional difference in COVID-19 mortality.	Ecological study utilizing a systemic review of literature	The findings revealed that four predictors explain regional differences in COVID-19 mortality. These predictors are severity of infection, number of elderly living in assisted facilities, population density, and standard rate of diabetes. Predictors could explain over 95% of the differences in cross-regional mortality observed in Italy from the onset of the epidemic to late 2020.	Possible bias from unpublished articles used in the systemic review. Also most models used for COVID-19 were undergoing peer review process when this systemic study began.
Goyal et al. [2]	No study was carried out. The article provided proposed guidelines on how to screen for and treat hyperglycemia in individuals with COVID in low-income settings.	This commentary proposed strategies for screening hyperglycemia in low-resource healthcare settings.	Reviewed guidelines on diabetes and proposed an algorithm based on simple measures of blood glucose (BG) to be implemented by healthcare workers in low-resource settings.	Goyal et al. mention that algorithm may help in early recognition of hyperglycemia and preventions of subsequent complications of COVID-19 [2].	Capillary testing may suffer from inaccuracy, especially at extremes of blood glucose values.
Gutierrez et al. [15]	Individuals tested for SARS-CoV-2 in Mexico	Assess non-communicable diseases, such as diabetes, and socioeconomic status as risk factors for COVID-19 severe outcomes.	Using data from national reporting of SARS-CoV-2 tested individuals in Mexico, they estimated the odds of hospitalization, intubation, and death based on pre-existing non-communicable diseases and socioeconomic indicators.	Obesity, diabetes, and hypertension were associated with increased rates of hospitalization, intubation, and death in individuals diagnosed with COVID-19. The poverty level is also associated with hospitalization and death.	Poverty's association with reduced healthcare access may have underestimated the association between poverty and COVID-19 prognosis.
Saeed et al. [3]	260 Indian residents 30+ years old	To assess and enhance the knowledge, attitudes, and practices that are critical to managing the COVID-19 pandemic successfully, especially in high-risk groups, such as people with hypertension, diabetes, renal/respiratory disorders, etc.	A ​​cross-sectional online survey was carried out among 260 Indian residents 30+ years old. A structured questionnaire was converted into a Google form for online data collection. The questionnaire included collecting data about demographic details of participants, their knowledge about COVID-19, safe practice measurements, their comorbidities, and challenges faced by them in management.	Participants 50 years or older reported difficulty monitoring their health along with difficulty accessing medicine during the COVID-19 pandemic. Participants suffering from multiple comorbid conditions had difficulty accessing healthcare. Accessibility to medicine was significantly associated with the education of the participant. Also, access to lab investigations and medicines was found to be most difficult for diabetic participants.	Study may be limited by voluntary response bias.
Kalyanaraman et al. [19]	No study carried out. Article reviewed the fundamental mechanisms responsible for disparate underlying conditions related to the disproportionate impact of SARS-CoV-2 on ethnic minority groups, focusing on neutrophil extracellular traps (NETs), especially in African American populations.	To understand the hypothetical mechanisms for enhanced vulnerability of African Americans to SARS-CoV-2 infection, COVID-19 severity, and increased deaths.	Review article using a systematic approach to address the fundamental mechanisms responsible for disparate underlying conditions related to the disproportionate impact of SARS-CoV-2 on ethnic minority groups, focusing on neutrophil extracellular traps (NETs).	Elevated levels of NETs are produced in the lungs of COVID-19 patients, leading to hyperinflammation and respiratory failure. NETs can be used as a marker to predict deadly effects caused by COVID-19. Increased NETs are seen in several chronic medical conditions that are more commonly seen in African Americans, such as obesity, hypertension, diabetes, and sickle cell disease, which can increase the severity of COVID-19. The article concluded diabetes is a key risk factor for developing severe COVID-19, and COVID-19 patients with this underlying condition are more likely to die of respiratory and cardiovascular complications.	Mechanism discussed is only hypothetical at the time of the article being written. Conclusions that were drawn only discussed the "potential" of neutrophil extracellular traps putting African American populations at a higher risk of mortality and morbidity of COVID-19.
van der Linden et al. [25]	US users of the G6 rtCGM (real-time continuous glucose monitoring) system who uploaded data before and during COVID-19.	To determine how time in range (TIR) of glucose control was affected during the COVID-19 pandemic.	Data analysis of app data from users of the G6 rtCGM system	The overall average TIR (time in range; i.e. of glucose control) improved by 0.3% during the early pandemic period. Higher COVID-19 mortality was associated with higher proportions of individuals experiencing TIR improvements of >5 percentage points.	Inability to quantify the populations' age distributions, diagnoses, racial/ethnic backgrounds, type of diabetes or comorbidities, medication regimens, or adoption of other diabetes-related technologies.
Selden et al. [20]	Data from MEPS of individuals who fell under CDC health risks for COVID-19, including extreme obesity, current smoker, 65+, diabetes, asthma, emphysema or other COPD, cancer, or coronary heart disease.	To determine the possible explanations for racial and ethnic disparities amongst COVID-19 patients who were hospitalized or died due to COVID-19	Systematic review	COVID-19 outcomes stem from structural racism on many levels, including income, education, health insurance, access to medical care, access to food, and health status.	Only examined the non-institutionalized community, i.e. those individuals who are not in elderly care facilities, correctional facilities, etc. This is a limitation because it is estimated that 42% of all COVID-19 fatalities have occurred among residents of nursing homes and long-term care facilities.
Gao et al. [5]	COVID-19 patients with possible risk factors such as hypertension, diabetes, obesity, chronic lung diseases, heart, liver, and kidney diseases, tumors, clinically apparent immunodeficiencies, local immunodeficiencies, such as early type I interferon secretion capacity, and pregnancy.	To review the current data on a comprehensive list of possible risk factors associated with COVID-19 severity.	Scoping review	Diabetes has been identified as one of the major risk factors of severe clinical course and prognosis of Covid-19 patients.	Some studies in the scoping review address general risk factors of COVID-19 development and others specifically focus on risk factors for severe COVID-19 leaving very little room to assess risk factors' chronological impact on disease severity.
Vahidy et al. [16]	COVID-19 Surveillance and Outcomes Registry in Houston, Texas	To assess the role that race and ethnic disparities play in likelihood of COVID-19 infection and evaluate the pathways that lead to differences in infection rates amongst socioeconomic groups.	Cross-sectional analysis of COVID-19 Surveillance and Outcomes Registry	Racial minorities including Hispanics and non-Hispanic black individuals are almost twice as likely to test positive for SARS-CoV-2 when compared to the non-Hispanic white population.	Does not include diabetic care.
de Souza et al. [27]	Brazilian Ministry of Health Database (SIVEP-Gripe). Sample size was 162,045. Period from February 26th to August 10th, 2020	To create a clinical profile that can be used to assist the decision-making of physicians regarding poor prognosis patients.	Analyze demographic data, clinical symptoms, and comorbidities	Higher hospitalization rate among males and older population, higher lethality among hospitalized patients. Numerous prevalent symptoms were also discovered that included cough and dyspnea.	Short study
Kyazze et al. [22]	COVID-19 patients with diabetes in Africa	To illustrate methods to optimize care in diabetics with COVID-19 in Africa and understand the correlation between DM and negative COVID-related outcomes.	Research studies that outline comorbidities associated with diabetes and COVID-19 and compile their results to create strategies to improve COVID-19 outcomes in these patients	Strong correlation with DM being a factor leading to death in COVID-19 patients	Study only targets one population.
Gopalan et al. [13]	Population belonging to low socioeconomic stratum (SES) in India.	To examine and discuss the impact of the lockdown in response to the COVID-19 pandemic on social, economic, health, and National Health Programs in India.	Literature review	Morbidity and mortality due to COVID-19 in India are largely attributable to co-morbid conditions like diabetes, hypertension, or cardiovascular disease [13]. Based on a prediction model, the duration of lockdown is directly proportional to the worsening of glycemic control in patients with diabetes as well as would increase diabetes-related complications.	
Chun et al. [4]	A study group of 10,069 individuals with type 1 and type 2 diabetes utilizing ICD10 codes. The control group of 50,587 individuals were selected with varying ages, gender, and region.	To determine the infection risk of COVID-19 in patients with diabetes.	Utilized population data from the national health information database (2015-2019) in Korea as well as the national health insurance service which maintains all medical information and demographics of the entire Korean population because of their compulsory social insurance system.	The results indicated that diabetics utilizing insulin therapy had a higher risk of contracting COVID-19 than those without diabetes.	
Clark et al. [26]	188 countries	To understand the number of individuals at an increased risk of severe COVID-19 and how it varies between countries in order to develop strategies to vaccinate and protect those at the highest risk.	Population data derived from the Global Burden of Diseases, Injuries, and Risk Factors Study (GBD) 2017 and UN population estimates for 2020. Included patients who have at least one condition labeled as cause for severe COVID-19 in current guidelines.	It was estimated that 1.7 billion individuals, comprising 22% of the global population, have at least one underlying condition that could increase their risk of severe COVID-19. The prevalence of one or more conditions was approximately 10% by age 25 years, 33% by 50 years, and 66% by 70 years, and similar for males and females. It was estimated that 23% of the global working-age population (15-64 years) have at least one underlying condition. Chronic kidney disease and diabetes were the most common conditions in this age range.	
Glennie et al. [29]	Canadian patients with T1DM and T2DM	To examine the benefits of using advanced glucose monitoring systems.	Systematic review looking at journals from CINAHL, Ovid MEDLINE ALL, Embase, and APA PsycINFO. Used controlled search phrases across databases and excluded animal-only records. Also incorporated survey data from various healthcare systems in Canada.	COVID-19 caused a large shift to virtual medicine in both persons with and without diabetes mellitus. Utilization of advanced glucose monitoring systems has improved remote glycemic control by enabling patients to make more informed decisions about their diabetic regimen and increasing awareness of how certain factors directly affect their glucose level. Overall, use of these monitors enabled better self-management of blood glucose. These systems can also be used to send data digitally.	

## Conclusions

Overall, there is mixed evidence on how diabetic management has been affected by the ongoing COVID-19 pandemic. Studies showed that patients with diabetes and those of lower SES were susceptible to external factors (e.g. lockdowns) that put them at greater risks associated with COVID-19 infection. This mixed evidence, as well as the gap in the literature pertaining to how SES impacts healthcare decisions among patients with diabetes, indicates the need for more research in order to identify the obstacles and risks that patients with diabetes could face with future pandemics. While there may be mixed evidence as to how the COVID-19 pandemic has impacted diabetes healthcare management, evidence still proves that those with co-morbid conditions, including diabetes, as well as those of lower SES, were at an increased risk of adverse outcomes secondary to COVID-19 infection. Therefore, the need to improve the public health infrastructure remains a priority to improve health outcomes even in the face of a pandemic.
